# The Role of Healthcare Professionals in Encouraging Parents to See and Hold Their Stillborn Baby: A Meta-Synthesis of Qualitative Studies

**DOI:** 10.1371/journal.pone.0130059

**Published:** 2015-07-08

**Authors:** Carol Kingdon, Emer O’Donnell, Jennifer Givens, Mark Turner

**Affiliations:** 1 School of Health, University of Central Lancashire, Preston, Lancashire, United Kingdom; 2 Department of Women’s and Children’s Health, Institute of Translational Medicine, University of Liverpool, Liverpool, United Kingdom; Harvard Medical School, UNITED STATES

## Abstract

**Background:**

Globally, during 2013 there were three million recorded stillbirths. Where clinical guidelines exist some recommend that professionals do not encourage parental contact. The guidance is based on quantitative evidence that seeing and holding the baby is not beneficial for everyone, but has been challenged by bereaved parents' organisations. We aim to inform future guideline development through a synthesis of qualitative studies reporting data relevant to the research question; how does the approach of healthcare professionals to seeing and holding the baby following stillbirth impact parents views and experiences?

**Methods/Findings:**

Using a predetermined search strategy of PubMed and PsychINFO we identified robust qualitative studies reporting bereaved parental views and/or experiences relating to seeing and holding their stillborn baby (final search 24 February, 2014). Eligible studies were English language, reporting parental views, with gestational loss >20weeks. Quality was independently assessed by three authors using a validated tool. We used meta-ethnographic techniques to identify key themes and a line of argument synthesis. We included 12 papers, representing the views of 333 parents (156 mothers, 150 fathers, and 27 couples) from six countries. The final themes were: "[Still]birth: Nature of care is paramount", "Real babies: Perfect beauties, monsters and spectres", and "Opportunity of a lifetime lost." Our line-of-argument synthesis highlights the contrast between all parents need to know their baby, with the time around birth being the only time memories can be made, and the variable ability that parents have to articulate their preferences at that time. Thus, we hypothesised that how health professionals approach contact between parents and their stillborn baby demands a degree of active management. An important limitation of this paper is all included studies originated from high income, westernised countries raising questions about the findings transferability to other cultural contexts. We do not offer new evidence to answer the question "Should parents see and hold their stillborn baby?", instead our findings advance understanding of how professionals can support parents to make appropriate decisions in a novel, highly charged and dynamic situation.

**Conclusions:**

Guidelines could be more specific in their recommendations regarding parental contact. The role of healthcare professionals in encouraging parents to see and hold their stillborn baby is paramount. Parental choice not to see their baby, apprehension, or uncertainty should be continuously revisited in the hours after birth as the opportunity for contact is fleeting and final.

## Introduction

Globally, there were 142 million recorded births in 2013[1]. For approximately three million of these mother and infant pairs the baby was recorded as stillborn[2]. International estimates suggest that more than 75 per cent of stillbirths occur in the developing nations of south Asia and sub-Saharan Africa[2]. In high-income countries around one in every 200 pregnant women reaching more than 22 weeks gestation will have a stillborn baby[3]. Uncertainty surrounds the extent to which rates of stillbirth may have declined in recent years, with international efforts currently in progress to standardise stillbirth reporting[4]. Irrespective of place and time, stillbirth is a profound human tragedy. The experience of stillbirth involves physical implications for the mother, together with intense grief and lasting psychological trauma for both parents and wider family[5,6]. Studies have shown that stillbirth is associated with anxiety, depression and post-traumatic stress disorder in mothers, couples, siblings and grandparents [7–10]. Emotional distress and grief are often intensified because there is little consensus of social norms when a baby is born dead. Consequently stillbirth has until recently been a little talked about and socially isolating event. The 2011 Lancet stillbirth series emphasised the unique status of stillbirth within medicine and highlighted how grief may be exacerbated by social stigma and the standard of care provided to parents[11]. The importance of appropriate and considerate parental care by health professionals at the time of stillbirth, in respect of seeing and holding the baby, is the focus of this paper.

Since 2009, a number of clinical guidelines for the management of stillbirth have been published by professional organisations including the United Kingdom’s Royal College of Obstetricians and Gynaecologists (RCOG)[12], the American College of Obstetrics and Gynaecology (ACOG)[13] and the Perinatal Society of Australia and New Zealand (PSANZ)[14]^.^ In the UK, national guidelines from the National Institute for Clinical Excellence (NICE) also include recommendations for psycho-social care following stillbirth [15,16]. These guidelines are principally based on quantitative evidence of what is known about care practices that can help bereaved parents cope at the time and in the years following a stillbirth. In the UK and Australia, there has been controversy arising from discordance between health professional’s guidance, clinical guidelines and public opinion canvased by bereaved parents’ organisations[17,18]. All guidelines should be subject to a continuous cycle of updating taking into account best available evidence.

The management of stillbirth is known to vary within organisations, between individuals and has been subject to change over time[19]. In the UK before 1970, parental contact with the stillborn was prohibited by health professionals in an attempt to reduce psychological trauma[20]. In 1985, taking into account new evidence, the RCOG’s guidelines were updated to recommend that parents of stillborn infants should be encouraged to have contact with their baby[21–24]. Current RCOG guidance published in 2010 places the emphasis on parents to express a desire to see or hold their baby[12]. Existing guidance does acknowledge that evidence in this area is limited[12–14]^)^ they also privilege quantitative evidence[25] and professional opinion. At the same time as there is anecdotal and research evidence that healthcare professionals find caring for families who experience stillbirth one of the more difficult aspects of their job[26–32].^)^ To date, guideline development has paid limited attention to qualitative studies of parental views and experiences, of which there has been an increasing number in recent years.

Traditionally qualitative studies have not featured in medicine’s hierarchies of evidence that are used in the formulation of clinical guidelines[12]. This is currently changing as a result of developments in qualitative research and evidence synthesis[33] including the publication of the first Cochrane qualitative evidence synthesis[34]. A shift is apparent in the most recent NICE guideline update published in December 2014. Taking into account quantitative and qualitative studies it recommends an experienced practitioner discusses with a woman whose baby is stillborn or dies soon after birth, and her partner and family, the option of one or more of the following: seeing a photograph of the baby; having mementos of the baby; seeing the baby; holding the baby[16]. This paper is a meta-synthesis of qualitative research studies intended to identify healthcare worker practices that parents’ value. At the outset the research question was; how does the approach of healthcare professionals to seeing and holding the baby following stillbirth impact parents views and experiences?

## Methods

The study design was a meta-synthesis using a pre-determined search strategy developed by all authors. There was no study protocol. Standardised protocol requirements and registries for qualitative synthesis do not currently exist, but are being discussed by members of the Cochrane Collaboration and World Health Organisation Department of Reproductive Health CerQUAL (Certainty of Qualitative Evidence) working group. Publication standards are now available for meta-narrative reviews, which were developed as part of the RAMSES (Realist and Meta-narrative Evidence-Synthesis: Evolving standards) project[35]. Meta-narrative, is one of the more recent approaches to evolve from the meta-synthesis tradition, which is distinctive in its inclusion of qualitative and mixed-method studies in the synthesis of different approaches to studying the same topic. The present review is a meta-synthesis not a meta-narrative review.

### Meta-synthesis

Meta-synthesis has been described as the qualitative equivalent to meta-analysis. In meta-synthesis the generic term ‘meta’ refers to the translation of studies into one another. There are a number of approaches[36–41] most of which originate from Noblit and Hare’s[42] development of meta-ethnography. Meta-synthesis involves systematic study selection and quality appraisal, the identification of initial concepts (from individual study findings), and a protracted process of reciprocal translation (comparison of accounts directly comparable) and refutational translation (comparison of accounts directly oppositional) in the development of a new, distinct line of argument with an emergent hypothesis that fits all the studies. As with meta-analysis, the scope and rigour of meta-synthesis reviews, means that there is a greater potential for them to influence policy and inform practice than for individual qualitative studies[43].

### Search strategy and selection criteria

The search strategy was designed to locate studies reporting parental views and experiences of seeing and holding their stillborn baby. The search was designed to locate any studies that might include qualitative data, including survey designs with open-ended questions inviting qualitative responses, mixed method studies, focus group and individual interview studies. The final search was completed on the 24 February, 2014. All electronic searches had English language and human subjects restrictions imposed. They used the key words covering the main search domains including “seeing” OR “holding” OR “contact” AND “perinatal death” OR “pregnancy loss” OR “fetal death” OR “stillborn” OR “stillbirth” AND “grief” OR “bereavement” OR “psychology”. Searches were conducted in PubMed and PsychINFO. A handsearch was then carried out using the references obtained from the relevant papers. Two authors (EO, JG) initially reviewed all of the included papers independently, then together with the lead author (CK) to reach a final agreement on inclusion by consensus.

**Fig 1:**
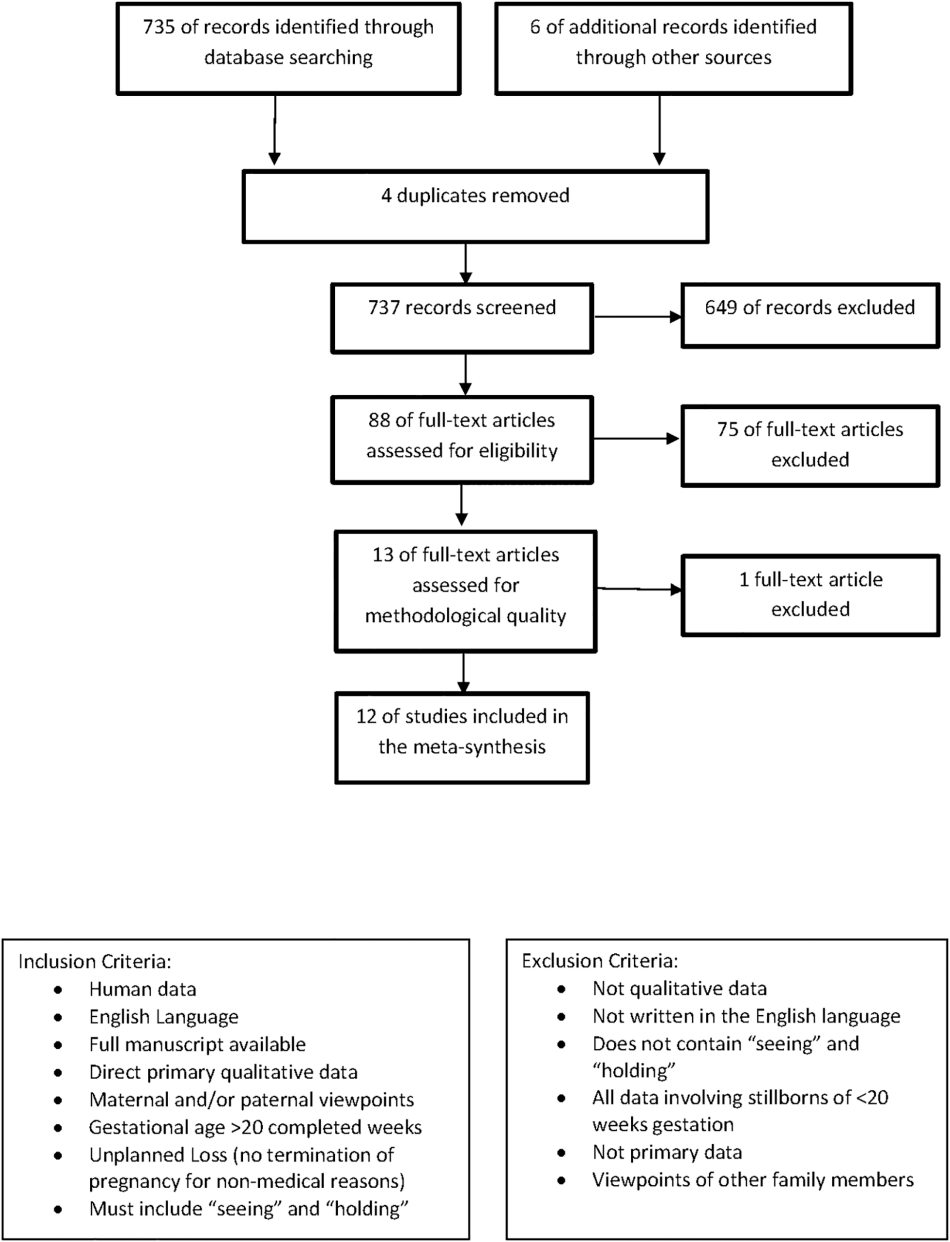
Process of article selection with inclusion and exclusion criteria.

Papers that included only maternal and/or paternal viewpoints were included, in accordance with the research question. All other family viewpoints were therefore excluded. No geographical criteria was placed on the search, or lower date restriction, as it has been shown that women’s memories of birth are generally accurate in following years and any memory lapses or confusion that can occur tend to be minor[44]. There is no standardised definition of stillbirth[4]. In the UK, stillbirth is defined by the Births and Deaths Registration Act 1953 section 41[45] (amended by the Stillbirth Definition Act 1992[46] as being: *“a baby which has issued forth from its mother after the 24* ^*th*^ *week of pregnancy and which did not at any time breathe or show any other signs of life”*. In Australia, stillbirth is defined as the death of a baby after 20 weeks in-utero until immediately before birth[47]. The World Health Organisation does not recognise a stillbirth until 28 weeks gestation[4]. Consequently we imposed the lower gestational limit of 20 completed weeks in utero. This encompasses the lower gestational limits referred to in current guidance. Articles reporting early miscarriages or termination of pregnancy for non-medical reasons were also excluded. Papers reporting miscarriage and/or termination of pregnancy that included data on stillbirth (>20 weeks gestation) reported separately were not excluded if they met all other inclusion criteria. Two papers fulfilled this criteria The full list of exclusion and inclusion criteria is shown in Fig 1 Process of article selection with inclusion and exclusion criteria.

### Quality assessment

Articles that had met the inclusion criteria were independently assessed by three authors (EO,JG, CK) to minimise bias. Quality appraisal was carried out according to a checklist described by Walsh and Downe[48] and articles were graded according to Downe and Simpson[49]. A grade of A was allocated to papers which had no or few flaws and D represented studies with significant flaws that would affect the credibility of the papers. All studies graded D were excluded. Any differences in the authors’ appraisals resulted in a re-read of that text and a decision was reached in unison by all three authors. The final grading is listed in Table 1: Summary of included studies.

**Table 1:** Summary of included studies.

Authors	Year	Location	Number of participants	Gestational Age	Length of time since Stillbirth	Method Used	Quality Grading
Lovell A	1983	UK	22 mothers10 stillbirths	20–27 weeks	Not stated	Interview	C-
Worth NJ	1997	Canada	8 fathers	26–41 weeks	3months-5years, 3 months	Interview	B
Samuelsson M, Radestad I, Segesten K	2001	Sweden	11 fathers	33–42 weeks	5–27 months	Interview	B-
Saflund K, Sjogren B, Wredling R	2004	Sweden	24 couples7 mothers	≥28 weeks	4–6 years	Interview	A
Trulsson O, Radestad I	2004	Sweden	12 mothers	≥24 weeks	6–18 months	Interview	B
Cacciatore J, Bushfield S	2007	USA	47 mothers	20–32 weeks (n = 13)33–36 weeks (n = 12≥37 weeks (n = 22)	Within 1 year (n = 10)1–2years (n = 10)2–5 years(n = 17)5–10 years (n = 7)≥10 years (n = 3)	Questionnaire	B+
Yamazaki A	2010	Japan	17 mothers	28–40 weeks	1–6 years	Interview	A
Cacciatore J	2010	USA	47 mothers	20–32 weeks (n = 13)33–36 weeks (n = 12)≥37 weeks (n = 22)	Within 1 year (n = 10)1–2years (n = 10)2–5 years(n = 17)5–10 years (n = 7)≥10 years (n = 3)	Questionnaire	B+
Lanthrop A, VandeVusse L	2011	USA	15 mothers	28–36 weeks	1–2 years (n = 5)2–4 years (n = 3)5–9 years (n = 7)	Interview	A+
Cacciatore J, Erlandsson K, Radestad I	2013	Sweden	131 fathers	>22 weeks	0–4 years (n = 99)5–10 years (n = 32)	Questionnaire	A
Lee, C	2012	Australia	14 mothers	20–24 weeks (n = 9)25–37 weeks (n = 4)1 non-responder	3–4 months	Questionnaire	B-
Downe S, Schmidt E, Kingdon C, Heazell AEP	2013	UK	22 mothers3 couples	24–42 weeks	1–9 years	Interview	A+

One paper[50] was discussed at length because it exhibited weaknesses resulting in a C grading. However the paper was published in 1983 when some of the quality measures on which it was judged were not common practice. For example, few Research Ethics Committees existed at that time and there were no standards for reporting methods of qualitative analysis. The final consensus was to include the study. A decision was also made to include four questionnaire studies containing free-text responses. In recent years the broad principles of qualitative thematic analysis have variously been applied to free-text responses in quantitative surveys of women’s and health professionals’ experiences of maternity care[31,51]. This extension of what is traditionally considered qualitative research is open to criticism on philosophical grounds with implications for quality appraisal. However, the design may allow opportunity to reach otherwise disengaged participants to provide a wider range of data, with participants providing detailed open-text responses.

### Analysis and synthesis

The analysis process began by identifying all relevant findings from one paper, and using them to generate a list of initial concepts[50]. These findings were then compared with the next paper and the list of initial concepts was added too. This process was repeated for all twelve papers to generate a single long list of initial concepts common to more than one paper. Next, these initial concepts were examined to identify similarities. This process is known as reciprocal translation whereby similar initial concepts are collapsed into emerging themes. Finally, three authors (CK, EO, JG) independently reviewed these themes before coming together and engaging in the process of refutational translation. In other words, to identify any inconsistencies and contradictions in the data that were at odds with the emerging themes and to revisit and refine those themes until all data was explained and accounted for. This process generated three final themes and our line of argument synthesis.

### Reflexive accounting

Reflexivity is the process associated with researchers' self-awareness of how they impact and transform the research they undertake[52]. It is a key methodological consideration in qualitative research studies. Reflexive accounting allows the reader of the final research product to assess the degree to which the prior views and experiences of the researcher may have influenced the design, data collection and data interpretation of the study or in this case, the synthesis of the findings of multiple studies[43]. The study was conceived with an informed knowledge of stillbirth and degree of professional distance, which arguably limited bias based on the teams own experiences. CK, a medical sociologist and an experienced maternity care researcher, conceived and designed the study with MT, a clinical academic and Consultant Neonatologist. CK’s prior knowledge of stillbirth was from undertaking primary research investigating midwives, obstetricians, perinatal pathologists and bereaved parents views and experiences in the UK. MT has extensive clinical experience of perinatal bereavement. As a Consultant Neonatologist, his experience is predominantly of postnatal deaths and some stillbirths. From the outset MT recognised the need for further research in relation to seeing and holding the baby following stillbirth to inform optimal clinical care practices. CK believed there was a need to identify and synthesise the findings from robust qualitative studies in this area, as a result of her involvement in two earlier studies of stillbirth[31,53]. One of these studies was a qualitative study[53] that met the inclusion criteria for this paper and was subject to the same rigorous quality assessment as all other included studies. EO and JG were fourth year medical students with little prior knowledge of the area. They had no prior personal experience of stillbirth and their professional knowledge stemmed from their Obstetrics and Gynaecology placements. To minimise bias all data regarding seeing and holding the stillborn was extracted from included studies by EO and JG. The generation of initial concepts was closely supervised by CK, before all three authors engaged in the cyclical processes of independent and collective reciprocal translation and refutational analysis.

## Results

### Search outcomes

The search strategy yielded 735 results containing quantitative, qualitative and mixed methods papers. This is shown in Fig 1: Process of article selection with inclusion and exclusion criteria. Six additional records were identified through other sources (hand searching and reference lists). A total of 737 records were screened with 649 exclusions by title or abstract. Eighty-eight full-text articles were assessed for eligibility with 75 excluded. The remaining 13 qualitative papers underwent critical appraisal, with one excluded due to the poor quality of provided methodology.

### Description of included studies

The twelve papers included in this meta-synthesis represent the views of 333 parents (156 mothers, 150 fathers and 27 couples) from six countries; UK (2)[50,53], USA (3)[54–56], Sweden (4)[57–60], Canada (1)[61], Japan (1)[62] and Australia (1)[63]. The sample size of individual studies ranged between eight and 131 participants. The gestational age of the baby at the time of stillbirth ranged from 20 weeks to 41 weeks, and the shortest time period since stillbirth was three months. Two papers included data from parents who had experienced a stillbirth more than 10 years ago[54,55]. Eight papers collected data using interviews either face-to-face or over the telephone[50,53,56–59,61,62] whilst the remaining four papers used questionnaires[54,55,60,63]. Half of the papers were published in the last 4 years[53–56,60,62]. Table 1 summarises the included studies.

### Description of the themes

Following analysis, seven emerging themes and three final themes were generated to describe the experience of stillbirth from the parents’ viewpoint and the role of healthcare workers in relation to seeing and holding. The initial concepts, emerging themes and final themes are summarised in Table 2. The final themes were: “[Still]birth: Nature of care is paramount”, “Real babies: Perfect beauties, monsters and spectres”, and “Opportunity of a lifetime lost.” Our line-of-argument synthesis highlights the contrast between all parents need to know their baby, with the time around birth being the only time memories can be made, and the variable ability that parents have to articulate their preferences at that time. Thus, we hypothesised that how health professionals approach contact between parents and their stillborn baby demands a degree of active management.

**Table 2:** Summary of initial concepts, emerging themes and final themes.

Initial Concepts	Relevant Papers	Emerging Themes	Final Themes
The experience of males vs females	55, 57	Nature of care during labour, birth and the immediate postnatal period has long-term consequences for bereaved parents’ wellbeing	Theme 1 **[Still]birth: Nature of care is paramount**
Healthcare professionals as equals	50, 56, 59		
Patronising attitudes of staff	50		
Impact of stillbirth on staff	50		
Lack of facilities after stillbirth	50, 63		
Positive attitudes of staff	50, 53, 54, 57, 58, 60, 63		
Staff providing opportunities	50,53,54,60,63	Provision of information, guidance, and encouragement by healthcare professionals is especially welcomed by parents literally at a loss about what to do when birth brings death	
Healthcare professionals providing information	50, 53,54,56,57,58,59,60		
Validation of stillborn baby	53,54,60,63	The importance of healthcare professionals acknowledging irrespective of gestation or condition a stillbirth is a baby	Theme 2 **Real babies: Perfect beauties, monsters and spectres**
Assumptive bonding	53,59		
Guidance from staff	57,58,60		
Spontaneous expression to see and hold stillborn baby	58,59,62	The actual and imagined appearance of a stillborn baby varies; Parents and professionals describe beautiful and perfect babies, damaged and/or deteriorating babies, and monsters, giving rise to spectres up until a baby is seen	
Appearance of stillborn baby	50,53,59,61,63		
Fear of meeting the stillborn baby	53,57,58,63		
Fear of judgment from staff	50,58		
Need for increased guidance	53,54,55		
Experience of seeing and holding	50,53,56,57,58,59,63	Experience of seeing and holding baby immediately after birth is the only opportunity parents have to cuddle, kiss, talk to, put a nappy on, bathe, dress or sleep alongside their child	Theme 3 **Opportunity of a lifetime lost**
Need for more time	53,55,58,59		
Regret	53,58,61	Regret, missed opportunities and need for more time	
Missed opportunities	50,54,55,63		
Lack of memories	54,55,61,62	Importance of memories and tokens of remembrance to grieve loss	
Preserving the memory of the stillborn baby	56,61,62		
Proof of existence	50,56		
Tokens of remembrance	50,56,58,60,62,63		

### Theme one: [Still]birth: The nature of care is paramount

Theme one incorporates two emerging themes describing staff actions and reactions before, during and after the labour.

#### The nature of care during labour, birth and the immediate postnatal period has long-term consequences for bereaved parents’ wellbeing

Small gestures such as staff talking to parents as they would to any other couple in their care makes the parents of stillborn babies look upon their experience in a more positive light. In seven studies[50,53,54,57,58,60,63] parents spoke warmly about the positive attitudes of the staff that cared for them.

*The staff made us feel like all other couples having a baby*.[60]

*She [the midwife] made me feel incredibly proud*. *A natural reaction after just giving birth is re-living the birth and wanting to talk about the birth experience*. *It sounds odd*, *but because I’d had such a good birth*, *I felt that I could behave like a normal mother*.[53]

Parents spoke about the importance of staff acknowledging them as parents of a baby and treating them as such. Furthermore, when staff appreciate that women are giving birth under difficult emotional circumstances, parents welcome the respect shown.

*I wasn’t just a woman giving birth*. *I was a woman giving birth under horrific circumstances*.[53]

An important theme across this data was the need to respect the memory of the stillborn baby. During the immediate post-natal period, parents showed great appreciation when staff handled and addressed their baby as ‘normal’. Data from four papers[53,54,60,63] suggested that when staff show respect to the stillborn baby, parents felt even more validated as parents.

*Our nurses called our baby by her name which helped our feelings to know that she was not being treated as another statistic*.[54]

Parents were also grateful when the midwives and doctors acknowledged the human gravity of their loss when delivering news and managing the stillbirth. Providing as much information as possible helps parents to understand the situation and begin to come to terms with the tragic circumstances they are facing.

*I thought the staff who took care of us were fantastic*. *They were people*, *not programmed machines in a huge organisation*. *People who dared to cry with us*, *who dared to stand by us in our pain and sorrow*. *Just totally fantastic*.[59]

*We need you to be real*, *and*, *you know*, *take off that white lab coat and become a human*.[56]

In contrast to staff treating parents as equals, in one study a mother described an encounter with a doctor who did not provide adequate information and treated her as if she would have trouble understanding the medical reasons as to why she had lost her baby.[50] Two other studies[57,63] reported that parents felt information could have been communicated better, with the use of complex medical terminology making it more difficult for them to understand what was happening.

*They treat you as if you’re a bit of a moron…she just told me not to eat green potatoes next time I get pregnant*.[60]

*We had no idea what the doctor was talking about as we had never heard of it [anencephaly]*. *All I remember the doctor saying to us was NOT COMPATIBLE WITH LIFE*. *[Emphasis in original]*.[63]

Other poor management of stillbirth included lack of facilities for women after having given birth to their stillborn babies. They felt their needs were not being met when placed on wards with other women who had just given birth. This heightened grieving in a highly emotional period of their lives.

*I know there really isn’t anywhere else for mums who have lost or are losing their babies but it really is awful to be listening to other peoples babies cry when your precious one has died*.[63]

#### Provision of information, guidance, and encouragement by healthcare professionals is especially welcomed by parents literally at a loss about what to do when birth brings death

When a piece of bad news is delivered, such as the diagnosis of stillbirth, it is difficult for parents to understand all information provided by staff. Patience and guidance from healthcare professionals are necessary for parents to process their situation and all of their options whilst simultaneously coming to terms with their loss. Comments about what parents valued included:

*They piloted us along*, *taking one step at a time*.[57]

*They talked with us and guided us through difficult questions*, *for example*, *concerning autopsy*, *about talking with a medical social worker and minister about how we were going to feel later*.[60]

In some cases, the guidance provided by staff was inadequate and parents expressed a need for increased information about opportunities for creating memories.

*I wish the nurses could have guided us more in the final hours with our son*. *I didn’t think to bathe him or dress him*, *or have our pictures taken holding him*. *I wish someone would have suggested it*.[54]

*No one told me I should bring a camera…no one told me that the baby would start changing colours*. *No one told me how hard it was going to be leaving the hospital without my baby*.[55]

The form of information and timing it is delivered is also of utmost importance during this period.

*She gave me a book*, *a parenting book*. *And she said “this is the only thing I can give you*. *The information is at the back and I didn’t have time to print it*.*” It was all about new parents*.[53]

*I cannot express how important it is for mums who go through a stillbirth to be given information immediately after it happens in order to help her (and husband) cope…being a physician myself*, *I asked to speak to a social worker on-call…she was less than helpful*.[54]

### Theme two: Real babies: Perfect beauties, monsters and spectres

A recurring theme across many quotes was the appearance of the baby and the worry of how others would react when parents decided to see the baby. Parents during this time felt a wide range of emotions, such as curiosity, fear of judgment from staff and fear ultimately of their reaction when first seeing their baby.

#### It is important for healthcare professionals to acknowledge that a baby born stillborn is still a baby, irrespective of gestation or condition

In three papers,[53,59,63] parents spoke about how staff overlooked the fact that the baby was stillborn and treated them as a living baby. This practice was greatly appreciated by parents and helped them to enjoy the experience of contact with their baby, rather than fearing it.

*They treated him as living baby*, *telling him how perfect and beautiful he was*. *They treated his body with respect and explained to him what they were doing*.[63]

*Even though she wasn’t breathing and she didn’t open her eyes*, *she still said “you’ve got a beautiful baby girl*.*” It just meant the world*.[53]

#### The actual and imagined appearance of a stillborn baby varies; Parents and professionals describe beautiful and perfect babies, damaged and/or deteriorating babies, which give rise to visualisations of monsters and imagined spectres until a baby is actually seen

When, or where contact with stillborn babies was not routinely encouraged, mothers allowed staff to decide whether their baby was fit to be seen. If the midwife described the baby in a positive manner, the mother would see them. When a baby was malformed or macerated and they were described negatively and un-baby like, parents would decline the opportunity for contact with their baby. The following quotes are from the UK in 1983.

*Your baby is perfect*, *you should see him*. *He’s beautiful…too beautiful for this world*.[50]

*Quite right*. *You wouldn’t like it*. *It’s an ugly little thing*.[50]

Having said that across time and place, until parents saw their baby, or to never see their baby, meant the baby was perceived as an entirely imagined being. As guidance has changed and it has become routine to provide the option of contact with the stillborn baby, more parents report the positive aspects of their baby’s appearance.

*To see his full head of hair and his eyes closed and five fingers*, *five toes*, *two ears*, *one nose*, *all the accessories*. *Everything was in perfect proportion*. *The baby*, *he was perfect*.[61]

*He had such long fingers*. *Yeah*, *that’s the kind of thing that stays with you*.[59]

In one study, where all participants were given the choice to see, hold and/or bathe their baby, one woman (the only one who did not give her baby a name) chose not to[63]. This may have been one situation where seeing and holding would not have been advantageous. Exactly how health professionals judge in which situations seeing and holding is unlikely to be advantageous was unclear, but our findings do suggest this requires considerable skill and knowledge. Evidence to guide professionals in relation to a certain gestation, malformation or time elapsed since death was not present. Five of the papers[50,56,61,62,63] did include details of the cause of death from which only an idea of a baby’s general appearance at birth can be construed. At least thirteen babies were known to have died from umbilical cord complications; placental abruptions (n = 2); anencephaly (n = 6); trisomy disorders (n = 6), fetal hydrops (n = 2); and “major foetal abnormalities” (n = 2). Parents expressed fear of meeting their baby in four studies,[53,57,58,63] but did not regret seeing and holding their baby when they did so.

*They wrapped Bill [the stillborn baby] in a blanket*. *We didn’t look at his body… just his little face… It was an amazingly good thing to have done*.[50]

*I had to take a couple of deep breathes before I dared to look at her*, *so I could get used to it slowly*.[59]

*I didn’t want to see Adam when he was immediately born due to his skull and brain missing*, *I was scared*. *I had to go for a D and C so before I went I wanted to see and hold him; the staff were great about this*. *We got to see him as much as we wanted*.[63]

### Theme three: *Opportunity of a lifetime lost*

Three emerging topics feature in this theme encompassing the experience of seeing the baby for the first time and the reflection on missed opportunities during what is a limited period of time to make as many tangible memories as possible.

#### The time immediately after birth is the only opportunity parents will ever have to cuddle, kiss, talk to, put a nappy on, bathe, dress or sleep alongside their child

Three papers[56,58,63] contained data describing what actions parents carried out when being with the baby for the first time. All were actions that they would have normally carried out with a new baby and would not be able to in the future.

*I slept with him*. *Just held him real close to me*. *Talked to him a lot*, *kissed him a lot*, *just tried to savour every moment with him I could*. *It was like trying to have a lifetime with him*.[56]

*When your baby dies…you’re not going to feed your baby and you’re not going to get to do all those things you do when you’re baby is healthy and you bring it home*. *So to give her a bath and dress her was really important to us*.[56]

Parents spent varying amounts of time with their infants depending on a number of factors, one being worry of staff perception and judgment.

*What influenced me was that I did not know for how long the staff thought it was OK to be with the baby and I was also afraid that the body would change*.[58]

### Parents can regret missed opportunities and wish they had more time

Reliving the experience gave the parents opportunity to reflect anything they would have done differently at the time. Regret was expressed in the form of missed opportunities, length of time spent with the baby and the lack of memories they are left with when it is no longer possible to create them.

Three studies reported parents regret at having decided not to hold their baby. Staff may need to offer the opportunity more than once so that parents understand that they may change their mind at any time.

*I wish someone had said to me in those first few hours*. *Even if you don’t want to see her now*, *you can see her in an hour or two*. *Or in a day or so…I was left to believe because I said I wasn’t ready to see her that was final*.[53]

*I regret not having held my baby and that’s the hardest thing*, *because I can’t change that…*[50]

One other regret that was expressed was the need for more time with the child. Some participants described feeling as though they were unwelcome and that staff wanted to discharge them from hospital as soon as possible rather than deal with them. This leads to a lack of memories that are very important to the parents.

*They only left him with me for about an hour*, *then they just took him away*. *I was begging them not to take my baby*.[53]

*They wanted to chuck me out as soon as I woke up on the Sunday*.[50]

### Importance of memories and tokens of remembrance to grieve loss

Memories and tokens of remembrance act as a tangible link to the baby who parents can no longer see. Tokens provide proof of existence and parenthood. Staff guidance in this area is necessary as many parents will not realise that they are able to carry out such activities, or comprehend the significance of mementos at the time. Two papers[58,59] report the value parents placed on siblings and grandparents also seeing and holding the baby.

*Since the time with the baby is too short anyway*, *you really need help with what to do with the baby*. *I think it is very important to have as many memories as possible so you can face the grief and be able to mourn properly*.[58]

*I’m so glad I have those pictures because otherwise*, *I’d think that really didn’t happen to me…that was just a bad dream*. *But the pictures are proof that the baby did exist*.[56]

*I think sometimes I wish I had held them both at the same time just to see what it felt like to have twins*.[63]

Tokens (footprints, handprints, hospital tags, blankets, toys) allow the preservation of the child’s memory and existence. The absence of memories and mementoes can result in difficulty grieving for the parents. Some parents expressed regret of not having sufficient tokens for remembrance.

*All I have now is this (the ultrasound picture)*. *I made a copy and put a cover over it so the colors won’t fade*.[62]

*If only I’d kept a lock of hair to prove I’d had someone*.[50]

*The hospital did up a box for us*, *with photos*, *foot and handprints*, *his little dress and a toy*. *I’m not really sure what else; I haven’t looked in the box*, *just not ready yet*.[63]

Even if parents did not ask for mementos to be prepared, parents valued this action in case they changed their preference at a later date. In the UK current professional guidance recommends this practice[12]. In one study of fathers, all participants maintained that photos should always be secured even if parents’ decline[57]. This practice is ethically justifiable in accordance with the principles of non-maleficence, beneficence, justice and autonomy. Even if parents initially decline respect for autonomy may be upheld as this practice gives parents the choice to obtain mementos at a later date. This is all the more important as the unique circumstances of stillbirth have been shown to impair parents’ ability to both articulate their preferences, and their competency to make decisions around the time of birth.

### Line of argument synthesis

Some current guidance for the management of stillbirth [12–15] recommends that healthcare professionals do not actively encourage contact with the stillborn baby but support any parent expressing the wish to do so. Our results suggest that healthcare professionals should actively inform parents of their options for contact with the baby following stillbirth and repeatedly offer these opportunities to parents in a way that is sensitive to each parent. As reported in Table 2, parents perceive an unmet need for increased guidance from staff [53,54,55], missed opportunities [53,55,58,59] and decisions made at the time giving rise to feelings of regret[53,58,61]. There is a contrast between the consistent need parents have to know their baby and the variable ability that parents have to articulate their preferences at the time of birth. As the time immediately after birth is the only time these memories can be made health professionals involvement and commitment to memory making is an essential component of appropriate and compassionate care.

### Hypothesis based on findings

This leads us to hypothesise that healthcare professionals should actively manage contact between parents and their stillborn baby. Active management should include judgments, based on the condition of the baby, the preferences of the parents and skilled support that provides information and options, as a prelude to choices. Healthcare professionals should tell parents about the opportunities to hold their baby. Some parents will benefit from encouragement to hold their baby. Exactly how much encouragement is exercised should be influenced by parents expressed preferences with the caveat that these can change and need to be revisited. Guideline authors should be more specific so that active management is guided by evidence of what was beneficial for other parents. Parents’ ambivalence and, or, fear should be actively negotiated as this is usually the only the time when parents have the opportunity to see and hold their stillborn baby. In other words, parental concerns should not be taken at face-value; professionals should explore what parents are concerned about, presenting facts and explanations that calm concerns, and taking into account the need for fluidity, develop a shared plan in the light of realistic expectations.

## Discussion

This paper sought to answer the question *how* does the approach of healthcare professionals to seeing and holding the baby following stillbirth impact on parents views and experience by meta-synthesising robust evidence from different qualitative studies, contexts and populations. We identified 12 studies, from six countries reporting parental views spanning four decades, the length of time since stillbirth ranged from three months to more than 10 years. Seven of the papers were published in or after 2007. The behaviour and opinions expressed by healthcare professionals were found to be especially pertinent in the decision making processes of parents. The nature and amount of care was paramount. Some parents did not feel able to express their desires to health professionals for contact with their baby, for contact over any great length of time, for repeated contact, or, to change their mind and request contact after an initial refusal. The possible time of contact is perilously short and should be used to create as many tangible memories as possible for the parents. When parents had a lack of mementoes, this was a source of grief and regret in the future. In some cases, staff had prepared such mementoes and kept them with notes, a practice which was gratefully acknowledged by many parents.

This paper’s originality lies in bringing together robust qualitative primary research studies in this area to offer new insights to inform practice. The synthesis has produced three distinct themes showing linkages between existing qualitative study findings, with added value in the weight this evidence provides over individual studies. The paper advances understanding of which practices parents of stillborn babies value to complement existing quantitative research. A 2012 review of the literature highlighted disparate results between quantitative studies of maternal psychological outcomes and parents holding their stillborn baby[64]. The author concluded it is not clear what parents should be advised as existing evidence is methodologically limited. This meta-synthesis does not offer new evidence to answer the question “Should parents see and hold their stillborn baby?” but addresses the more complex issue of “How can healthcare professionals support parents to make appropriate decisions in a novel, highly charged and dynamic situation?” The juxtaposition of our findings with existing quantitative research could lead to a richer and more nuanced understanding of the role of healthcare professionals in shaping parental experiences and long term wellbeing. A recent paper integrating disparate findings about miscarriage and women’s wellbeing demonstrates how contradictions between qualitative and quantitative findings have considerable value in provoking such a process and can lead to more sophisticated understandings of complex phenomena[65].

This study was stimulated by a discordance between professional guidance and a campaign by the UK’s Stillbirth and Neonatal Death Society (SANDS)[17]. Our results support the inclusion of suggestions from bereaved family support groups in professional guidelines. Specifically the detailed principles of good practice set out by SANDS[66] and similar organisations around the world [67,68], which resonate with many of the parental views included in this meta-synthesis. The evidence suggests a number of ways healthcare professionals can support parents to make appropriate decisions in a novel, highly charged and dynamic situation. For example, information should be spoken, written and revisited to ensure understanding if choices are to be made. The role of professional’s should encompass acknowledging the human gravity of the parents’ loss, at the same time as they address and handle the stillborn baby as they would a live baby; this not only validated them as parents but helped parents to begin to grieve. The evidence also suggests that parents particularly value professional guidance about exactly *how* to see and hold. This includes for how long, for whom else it may be beneficial (i.e. siblings and/or grandparents), how best to see and photograph (i.e. with a head covered, with one or more parents, as a family), what to expect if they want to bathe, dress or sleep next to their baby, and how the passing of time will alter the baby’s temperature, appearance, and touch. The appearance and feel of the stillborn baby was an important issue to parents; the unknown sometimes cause apprehension and fear. Parents who saw their baby described the ‘perfect’ parts of them and compared them with siblings or other family members. This process allowed bonding with the baby and further consolidated their existence.

The credibility of our findings is supported by a another recently completed comprehensive systematic review report addressing broader questions about families’ experiences and the appropriateness of interventions and strategies aimed at improving their psychological wellbeing following stillbirth[69]. This meta-synthesis specifically focused on seeing and holding. That review had a different question and scope; it includes any psycho-social interventions and strategies delivered or suggested by health professionals. In relation to parental contact, the qualitative review component also reports that information provision and guidance by health professionals to aid parental decision-making and prepare them for meeting their stillborn baby is key, encouragement or direction to assist parents how to hold their stillborn is important, and parents may later regret not having had contact even though they expressed no desire at the time. One of the main criticisms of qualitative research is that it is not generalizable. It is also characterised by fundamental differences in underlying epistemology and misconceptions derived from broader power imbalances between researchers[70]. Recent developments in qualitative evidence synthesis highlight the potential of qualitative research to provide robust evidence and inform guideline development[33,34]. This paper advances the case for the inclusion of qualitative synthesis in the guideline development process, clinical guidelines and hierarchies of evidence-based medicine more generally. The findings of the other meta-synthesis have already informed new guidance from the Stillbirth Foundation Australia, which includes ten detailed recommendations in respect of seeing and holding[67].

As highlighted in existing RCOG[12], ACOG[13], PSANZ[14] and NICE[15] guidance and evident in our second theme *‘Real babies*: *Perfect beauties*, *monsters and spectres’* seeing and holding the baby may not be advantageous for everyone. This paper does not answer the question “In what circumstances is seeing and holding advantageous?” or the related question “In what circumstances is seeing and holding not advantageous?” Our findings do not run contrary to quantitative studies suggesting contact with the stillborn baby can be a positive experience for parents, and they help contextualise quantitative studies reporting possible adverse outcomes for mothers[25,71–75]. We recommend that how healthcare professionals approach contact between parents and their stillborn baby demands a degree of active management. We offer new weight of evidence to inform more prescriptive guidance taking into account the tension between all parents need to know their baby and many parents inability to articulate clear preferences at the time of birth. It may be difficult to incorporate our findings into professional guidelines. The greater parts of most professional guidelines are made up of prescriptive statements that can be audited. In contrast we advocate a framework for judgments made by healthcare professionals as they work with bereaved families. This area of practice should be based on the judgments of healthcare professionals. We highlight evidence that supports the need for judgments and informs how those judgments are framed.

The methodological strengths of this paper include the use of a predetermined search strategy, quality assessment and systematic synthesis (S1 Checklist). Three study authors were involved in the identification of initial concepts, and reciprocal and refutational translation of themes to reduce bias. Both established and more recent approaches to qualitative and mixed-method synthesis [35,41] offer valuable research tools to summarise heterogeneous literatures and illuminate complex topic areas in new ways. Ongoing efforts for increased methodological transparency can only serve to increase their influence as clinicians, academics and policy makers increasingly engage with multiple and mixed methodologies. One important limitation of this paper is that all of the included studies originate from high income, westernised countries, raising questions about the transferability of our findings to other cultural contexts. The restrictions we imposed limited our sample to English language papers. This paper is also limited in the kinds of questions it can answer. For example, exactly *how* health professionals judge which situations seeing and holding is unlikely to be advantageous remains unanswered, but our evidence does show this requires considerable skill and clinical judgement, coupled with detailed knowledge of what has been beneficial to other parents in the past. Existing studies of professional views and experiences show they find caring for families who experience stillbirth one of the more difficult aspects of their job[26–32]. One UK national survey [31] and a more recent Irish qualitative study [32] report the urgent need for more formal training in bereavement care to support staff to improve their knowledge and ability to guide and support parents. This meta-synthesis adds to that evidence and should be used alongside quantitative study findings and family support group literature [66–68] in the development of comprehensive training tools for early-career obstetrician gynaecologists, midwives and nurses.

## Conclusions

Parental contact with their stillborn baby is an emotive issue. The role of healthcare professionals in encouraging parents to see and hold their stillborn baby is paramount in the short time-frame surrounding birth. Where parents’ express an initial preference not to see their baby, apprehension, or uncertainty about holding their baby, this decision should be revisited in the hours after birth. The opportunity for contact is fleeting and final.

## Supporting Information

S1 PRISMA Checklist (PDF)
